# Morphological and chemical characterizations of jujube (*Ziziphus jujuba* Mill.) to select superior accessions

**DOI:** 10.1002/fsn3.2831

**Published:** 2022-03-21

**Authors:** Ali Khadivi, Fatemeh Beigi

**Affiliations:** ^1^ Department of Horticultural Sciences Faculty of Agriculture and Natural Resources Arak University Arak Iran

**Keywords:** breeding, chemical properties, gene pool, jujube, yield

## Abstract

The fruits of jujube (*Ziziphus jujuba* Mill.) are consumed worldwide as food and herbal medicine because of their impact on human health and benefits. Here, phenotypic and chemical variation of this species was investigated to select superior accessions. The selected accessions showed significant differences based on the measured characteristics. Fresh fruit weight varied from 2.72 to 6.42 g with an average of 4.54, while dry fruit weight ranged from 0.89 to 2.57 g with an average of 1.55. Total phenolic content ranged from 1.69 to 14.05 mg gallic acid equivalent (GAE) g^−1^ fresh weight (FW) and total flavonoid content varied from 0.25 to 2.01 mg quercetin equivalents (QE) g^−1^ FW. Total anthocyanin content varied from 5.98 to 76.32 µg CyE g^−1^ FW. Radical scavenging activity (2,2‐diphenyl‐1‐picryl‐hydrazyl‐hydrate [DPPH]) ranged from 1.32 to 5.82 mg ascorbic acid equivalents (AsAE) g^−1^ FW, while ferric reducing antioxidant power (FRAP) varied from 35.37 to 93.35 µM FeSO_4_. The present study showed high diversity in morphological and chemical properties of jujube accessions. Based on the traits related to fruit quality such as fruit weight, fruit skin color, and fruit flavor, as well as in terms of chemical characteristics related to medicinal properties such as total anthocyanin content and antioxidant activity, 13 accessions were superior and are recommended to use in breeding programs. The commercial orchard of those best accessions should be extensively constructed to take advantage of the high yield of this tree as a crop and its medicinal properties.

## Graphical abstract

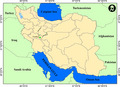

## 1 INTRODUCTION

Although jujube (*Ziziphus jujuba* Mill., the Rhamnaceae family) is mainly adapted to temperate and subtropical regions and naturally to areas with cold winters and hot summers, it is widely distributed in dry climates (Gao et al., 2011). Jujube fruit is rich in vitamins A, B, and C, as well as minerals and various compounds such as alkaloids, flavonoids, sterols, tannins, saponins, and fatty acids. Jujube also has significant antioxidant properties that can neutralize the activity of free radicals, so its fruit is involved in traditional medicine (Tatari et al., 2016). The flesh of jujube fruit contains protein, fat, carbohydrates, calcium, phosphorus, and iron (Karakaya et al., 2020; Ozturk et al., 2021). Jujube fruits are consumed worldwide as food and herbal medicine because of their impact on human health and benefits (Chen et al., 2017). Different parts of jujube are rich in medicinal properties such as antiseptic, analgesic, and antidiabetic. Jujube seeds are used because of their effect on reducing insomnia and anxiety. Jujube is used as a blood purifier, nerve sedative, stomach tonic, laxative, and antitussive (Gao et al., 2011).

Genetic diversity is the basis of agricultural programs and development. Genetic resources are the primary basis for creating promising improved cultivars, which today are considered to be the most valuable national resources and primary resources of any country (Behera et al., 2008). Gene banks are created to collect genetic material to examine genetic diversity and long‐term protection. Materials collected in terms of population, species, and gene pool need to be diverse. In any country, it is essential to study the genetic diversity of plant species to use them in breeding, conservation, management, and establishment of plant species (Ghazaeian, 2015). Iran is one of the richest countries in the world in terms of plant genetic resources. The country has an advantage in terms of plant diversity, which is part of the existing biological richness, due to exceptional ecological characteristics (Khadivi, 2018).

Morphological characterization is one of the first steps to identify plant genetic resources. Evaluation of morphological traits, genetic resources, and the collection of desirable traits in one cultivar is one of the important breeding goals in plants (Khadivi‐Khub & Anjam, 2014). Also, knowledge about the chemical properties of a plant species can help the food and pharmaceutical industries. Superior accessions can be used to produce different food, pharmaceutical, and antioxidant products (Halliwell & Gutteridge, 1990). The present research aimed to investigate the phenotypic and biochemical characterizations of jujube (*Z. jujuba*) and then to select superior accessions for cultivation in the orchards as well as use in future breeding programs.

## 2 MATERIAL AND METHODS

### 2.1 Plant material

**FIGURE 1:**
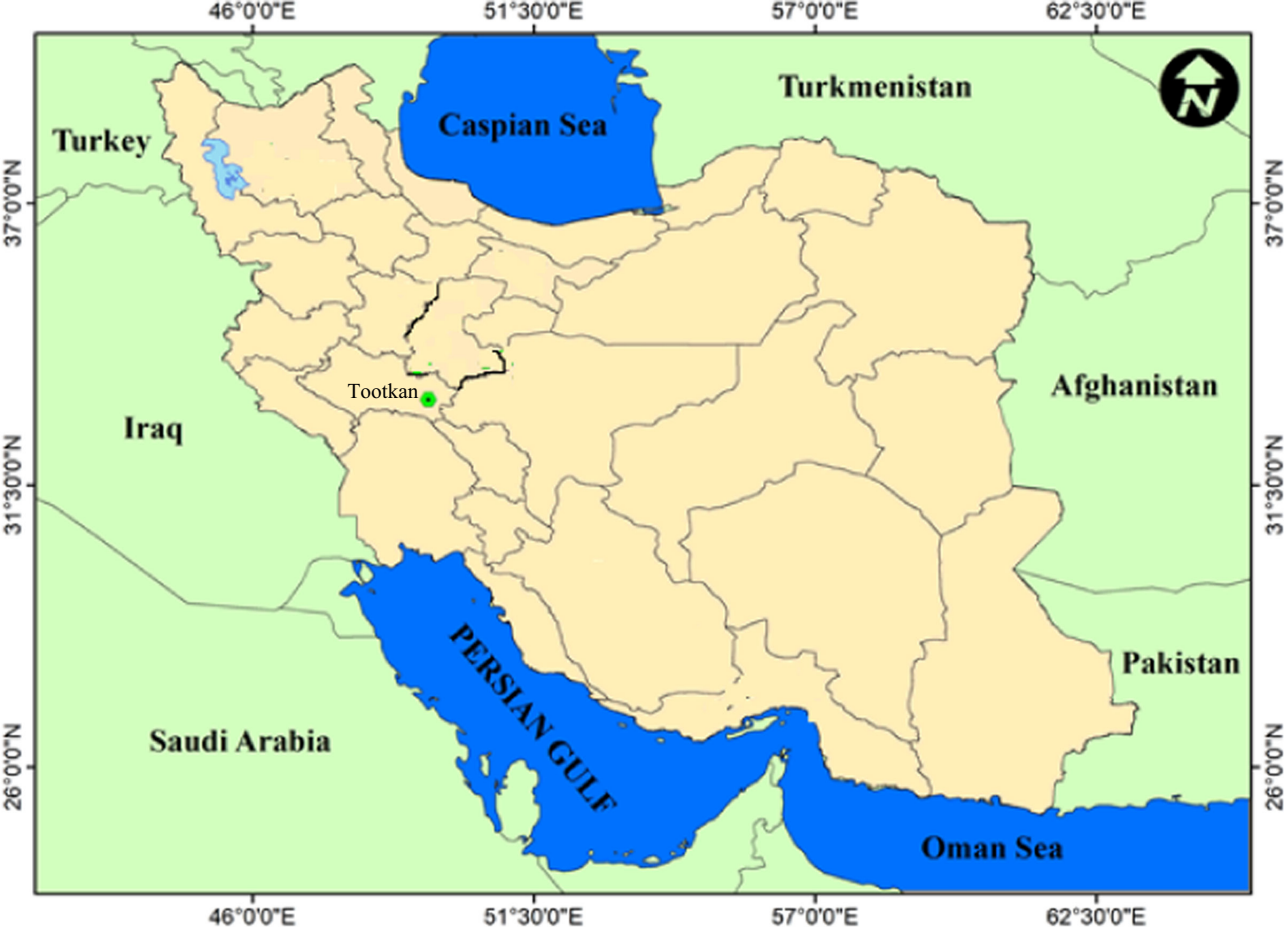
Geographic location of collection site for the studied accessions of *Z. jujuba*

In the present study, 100 local accessions of jujube (*Z. jujuba*) in a collection site from Tootkan area in Lorestan province/Iran were selected. The sampled accessions were chosen randomly based on their health and yield. Tootkan area is located at 32°54′57″N latitude, 49°39′31″E longitude, and 2025 m height above sea level (Figure 1).

### 2.2 Morphological and pomological analysis

Phenotypic diversity of the accessions studied was investigated using 39 morphological traits according to the International Plant Genetic Resources Institute (IPGRI) guideline (Saha, 1997). The 50 mature leaves and 50 mature fruits in each accession were used to record the related characters. The length and width of leaf, fruit, and stone were measured using a digital caliper. Also, fresh fruit weight, dry fruit weight, and stone weight were measured using an electronic balance with 0.01 g precision. Furthermore, the qualitative characters were estimated based on rating and coding (Table 2) (IPGRI, Saha, 1997).

### 2.3 Chemical analysis

#### 2.3.1 Total phenolic content

For extraction, samples (1.00 g) were homogenized using 10.00 ml of 80.00% methanol, and the mixtures were centrifuged at 4472 *g* (revolutions per minute) for 10 min. Supernatants were collected and analyzed for total phenolic content and antioxidant activity assays. Total phenolic content of fruit extracts was measured using the Folin–Ciocalteu reagent method with spectrophotometry (Singleton & Rossi, 1965). Briefly, 400 μl of the extract was combined with 2.00 ml of 10‐fold diluted Folin–Ciocalteu reagent and 1.60 ml of sodium carbonate 7.50% and then placed at room temperature for 30 min. The absorbance was estimated at 756 nm. The concentration of total phenolic content was read in mg gallic equivalents per g fruit weight (FW) using a calibration curve prepared with gallic acid.

#### 2.3.2 Total flavonoid content

For determination of total flavonoid content, the method described by Grzegorczyk‐Karolak et al. (2015) was adopted so that the 2 ml of fruit extracts was mixed with 2 ml of 2.00% aluminum chloride (AlCl_3_) and the reaction mixture was allowed to stand for 15 min at room temperature. The absorbance was measured at 415 nm, and the findings were expressed as mg quercetin equivalents per g FW (mg QE/g FW) for total flavonoid content.

#### 2.3.3 Total anthocyanins

Total anthocyanins in fruits were extracted with mixing 0.50 g of fresh materials with 10 ml of acidified methanol containing 1% HCl (v/v). The extract was centrifuged at 4472 *g* for 10 min and the absorbance of supernatants was recorded at 530 nm (Nogues & Baker, 2000). The content of anthocyanins in fruit samples was calculated using the extinction coefficient of cyanidin‐3‐glucoside (cyd‐3‐glu) and expressed as mg Cyd‐3‐glu equivalents.

#### 2.3.4 Radical scavenging activity

The scavenging activity of the extracts prepared on 2,2‐diphenyl‐1‐picryl‐hydrazyl‐hydrate (DPPH) free radicals was determined. The 25 μl of the fruit extract was reacted with a 0.10 mM methanol solution of DPPH in a total volume of 3.00 ml, and the mixture was then placed in the dark at room temperature for 30 min. The absorbance was read at 517 nm. The DPPH scavenging activities were calculated based on the following formula:

$$\text{DPPH}\;\text{scavenging}\;\text{effect}\;\left(\%\right)=\left(A_{\text{control}}‐A_{\text{sample}}/A_{\text{control}}\right)\times 100.$$

Where *A*_control_ and *A*_sample_ represent the control absorbance and the sample absorbance, respectively (Zhu et al., 2009). The DPPH scavenging activity of fruits was expressed as mg ascorbic acid equivalents (ASAE) per g FW using the established ascorbic acid calibration curve.

#### 2.3.5 Ferric reducing antioxidant power (FRAP)

The method developed by Benzie and Strain (1996) was used for the FRAP assay. The FRAP reagent comprised of 300 mM acetate buffer, 10 mM TPTZ (2,4,6‐tripyridyl‐s‐triazine) in 40 mM HCl and 20 mM ferric chloride (10:1:1, v/v/v). To 20 μl of fruit extract was added 3.00 ml of FRAP reagent, and the reaction mixtures were placed in a 37°C water bath for 10 min. The absorbance was read at 593 nm, and antioxidant activities were determined using the prepared FeSO_4_ standard curve.

### 2.4 Statistical analysis

The Spearman correlation coefficient with SPSS software (PSS Inc., Norusis, 1998) was applied to determine the simple correlations between the traits. Principal component analysis (PCA) was used to study the relationships among the accessions with SPSS software. Also, the scatter plot was created using the first three principal components (PC1/PC2/PC3) with SPSS software. The Ward's method and Euclidean distance were used to hierarchical cluster analysis (HCA) with PAST (PAleontological STatistics) software (Hammer et al., 2001).

## 3 RESULTS AND DISCUSSION

### 3.1 Morphological descriptions

Out of 39 morphological traits measured, nine characters, including branch color (gray in all the accessions), leaf density (high), leaf shape (ovate), leaf apex shape (obtuse), leaf upper surface color (dark green), leaf lower surface color (light green), leaf serration (present), fruit shape (oval), and fruit stone surface (coarse), had no differences among the studied accessions. Thus, they were excluded from analysis and Tables. The morphological traits without variation are more homogeneous and repeatable among the accessions, and therefore may be considered as stable traits. The remaining 30 morphological characters and also all five chemical properties had high variabilities. The highest CV (400.00%) belonged to thorn presence on annual shoot (Table 1).

**TABLE 1:** Descriptive statistics for morphological and chemical characters utilized in the studied accessions of *Z. jujuba*

No.	Character	Abbreviation	Unit	Min	Max	Mean	*SD*	CV (%)
1	Tree growth habit	TGH	Code	1	5	1.56	1.21	77.56
2	Tree growth vigor	TGV	Code	3	5	4.86	0.51	10.49
3	Tree height	TH	Code	3	5	4.92	0.39	7.93
4	Trunk type	TrTy	Code	1	3	1.14	0.51	44.74
5	Canopy density	CD	Code	3	5	4.76	0.65	13.66
6	Branching	Bra	Code	3	5	4.90	0.44	8.98
7	Branch density	BrD	Code	3	5	4.90	0.44	8.98
8	Branch flexibility	BrF	Code	1	3	1.02	0.20	19.61
9	Annual shoot length	AnBrLe	cm	11	105	19.99	12.09	60.48
10	Thorn on annual shoot	ThoBr	Code	0	1	0.06	0.24	400.00
11	Leaf length	LLe	mm	36.44	54.43	44.11	4.23	9.59
12	Leaf width	LWi	mm	18.46	28.82	21.13	2.56	12.12
13	Petiole length	PeLe	mm	2.50	7.32	3.26	0.78	23.93
14	Petiole thickness	PeThi	mm	0.44	2.18	1.18	0.35	29.66
15	Ripening date	RiDa	Date	Mid‐Sep	Late‐Sep	2.40	0.92	38.33
16	Yield	Yi	Code	3	5	4.96	0.28	5.65
17	Fruit length	FrLe	mm	21.96	29.45	24.78	1.49	6.01
18	Fruit width	FrWi	mm	16.59	23.89	19.02	1.09	5.73
19	Fresh fruit weight	FreFrWe	g	2.72	6.42	4.54	0.70	15.42
20	Dry fruit weight	DrFrWe	g	0.89	2.57	1.55	0.31	20.00
21	Fruit stalk length	FrStLe	mm	1.54	4.58	2.38	0.49	20.59
22	Fruit stalk diameter	FrStDi	mm	0.58	3.33	1.05	0.30	28.57
23	Fruit flesh thickness	FrFlThi	mm	4.19	10.09	5.66	0.77	13.60
24	Color of fruit skin	FrSkCo	Code	1	3	2.98	0.20	6.71
25	Color of fruit flesh	FrFlCo	Code	1	3	2.98	0.20	6.71
26	Fruit flavor	FrTa	Code	1	3	1.08	0.39	36.11
27	Texture of fruit flesh	FrFlTex	Code	1	3	2.66	0.76	28.57
28	Length of fruit stone	FrStLe	mm	13.41	19.23	16.12	0.98	6.08
29	Width of fruit stone	FrStWi	mm	6.91	11.48	7.56	0.47	6.22
30	Weight of fruit stone	FrStWe	g	0.31	0.47	0.40	0.04	10.00
31	Total phenolic content	TPC	mg GAE g^−1^ FW	1.69	14.05	5.65	2.38	42.12
32	Total flavonoid content	TFC	mg QE g^−1^ FW	0.25	2.01	0.83	0.19	22.89
33	Total anthocyanin content	TAC	µg CyE g^−1^ FW	5.98	76.32	46.55	14.52	31.19
34	Radical scavenging activity	DPPH	mg AsAE g^−1^ FW	1.32	5.82	3.34	0.81	24.25
35	Ferric reducing antioxidant	FRAP	µmol FeSO_4_ g^−1^ FW	35.37	93.35	59.69	13.16	22.05

The accessions showed three types of growth habit, including spreading (80 accessions), semi‐erect (12), and erect (8). Tree growth vigor, tree height, canopy density, branching, and branch density were predominantly high (Table 2). In general, the higher tree growth vigor, the higher tree height, branching, and branch density, and as a result, the fruit‐related traits will be more desirable, and thus the yield, fruit length, and dry fruit weight are increased (Hosseini et al., 2018).

**TABLE 2:** Frequency distribution for the measured qualitative morphological characters in the studied accessions of *Z. jujuba*

Character	Frequency (no. of accessions)			
	0	1	3	5
Tree growth habit	–	Spreading (80)	Semi‐erect (12)	Erect (8)
Tree growth vigor	–	–	Intermediate (7)	High (93)
Tree height	–	–	Intermediate (4)	High (96)
Tree trunk type	–	Single‐trunk (93)	Multitrunk (7)	–
Density of canopy	–	–	Moderate (12)	High (88)
Branching	–	–	Intermediate (5)	High (95)
Branch density	–	–	Intermediate (5)	High (95)
Branch flexibility	–	Low (99)	Intermediate (1)	–
Thorn on annual shoot	Absent (94)	Present (6)	–	–
Ripening date	–	Mid‐Sep (30)	Late‐Sep (70)	–
Yield	–	–	Intermediate (2)	High (98)
Fruit skin color	–	Brown‐red (1)	Brown (99)	–
Fruit flesh color	–	Cream (1)	Light green (99)	–
Fruit taste	–	Sour‐sweet (96)	Sweet (4)	–
Fruit flesh texture	–	Soft (17)	Firm (83)	–

The range of quantitative leaf‐related characters was as follows, leaf length: 36.44–54.43 mm, leaf width: 18.46–28.82 mm, petiole length: 2.50–7.32 mm, and petiole thickness: 0.44–2.18 mm (Table 1). In the study of a jujube collection from the north of Iran, the ranges of leaf length, leaf width, and petiole length have been reported as 25.00–56.00 mm, 13.60–24.60 mm, and 0.20–4.10 mm, respectively (Ghazaeian, 2015).

The fruits in the majority of accessions were ripened in late‐September (70 accessions). Most of the accessions (98 out of 100) showed a high yield. Fruit skin color was brown in 99 accessions, and also fruit flesh color was light green in 99 accessions (Table 2). In the study of a jujube collection from Ukraine, skin color showed strong diversity, ranging from brown‐yellow to dark brown (Grygorieva et al., 2014).

Fruit taste was sour‐sweet in 96, and also sweet in four accessions. The range of quantitative fruit‐related characters was as follows, fruit length: 21.96–29.45 mm, fruit width: 16.59–23.89 mm, fruit flesh thickness: 4.19–10.09 mm, fruit stalk length: 1.54–4.58 mm, and fruit stalk diameter: 0.58–3.33 mm. Also, fresh fruit weight varied from 2.72 to 6.42 g with an average of 4.54, while dry fruit weight ranged from 0.89 to 2.57 g with an average of 1.55 (Table 1). In the study of a jujube collection from the north of Iran, the ranges of fruit length, fruit width, and fruit weight have been reported as 14.60–21.30 mm, 15.30–21.60 mm, and 0.79–4.80 g, respectively (Ghazaeian, 2015). Also, the range of 0.14–6.33 g has been recorded for fruit weight in jujube from China (Liu et al., 2009).

**FIGURE 2:**
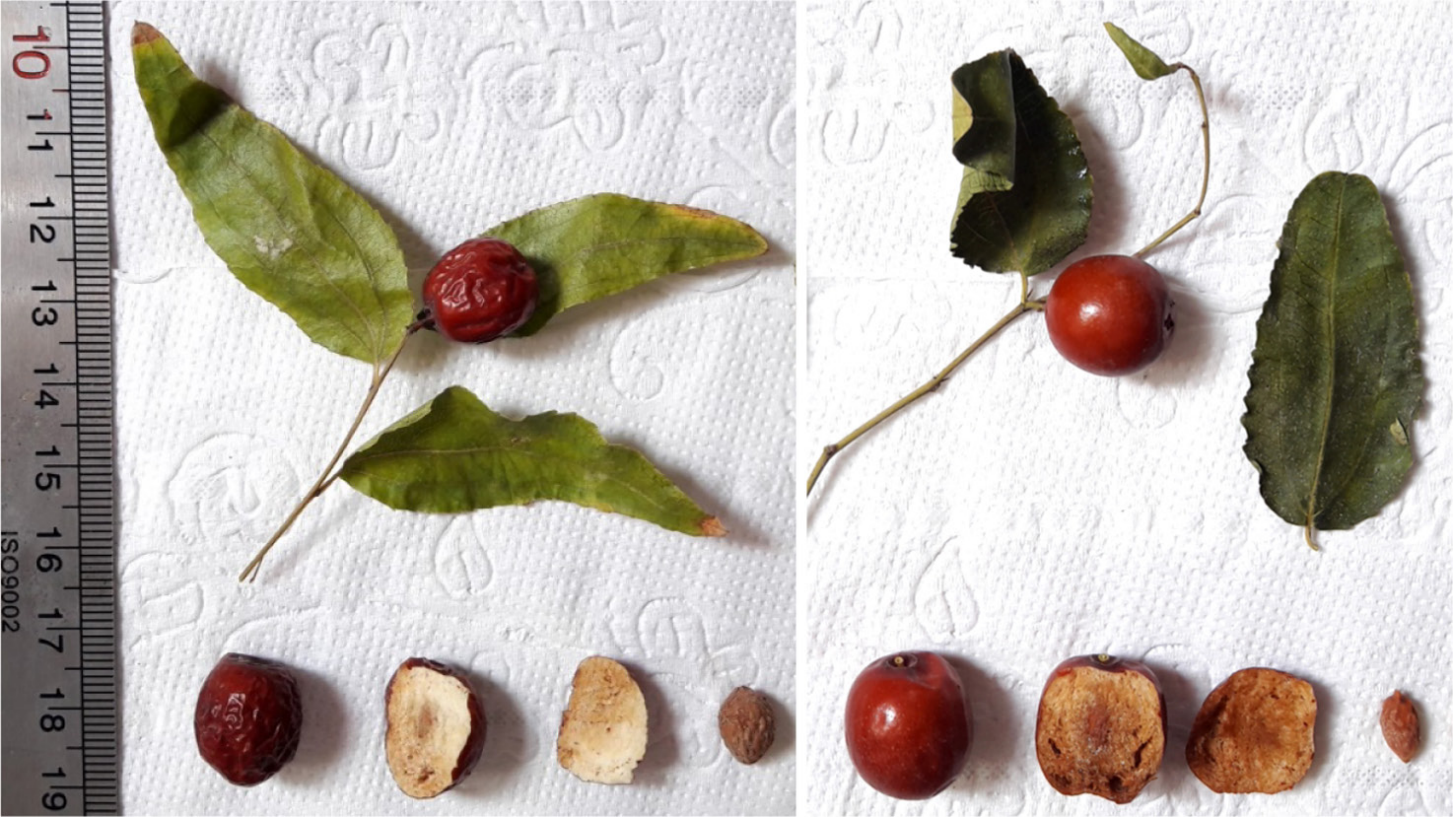
The leaf and fruit's pictures of two accessions studied of *Z. jujuba*

Stone length ranged from 13.41 to 19.23 mm, stone width varied from 6.91 to 11.48 mm, and stone weighed from 0.31 to 0.47 g (Table 1). In the study of a jujube collection from the north of Iran, the ranges of stone length, stone width, and stone weight have been reported as 10.20–13.50 mm, 3.80–7.90 mm, and 0.26–1.93 g, respectively (Ghazaeian, 2015). Besides, in the study of a jujube collection from Ukraine, the ranges of stone length and stone width have been reported as 12.84–28.67 mm and 5.06–9.74 mm, respectively (Grygorieva et al., 2014). Also, the ranges of 0.28–0.65 g (Sivakov et al., 1988) and 0.06–1.90 g (Ghosh and Mathew, 2002) have been recorded for stone weight in jujube collections from different countries. The leaf and fruit's pictures of two accessions studied of *Z. jujuba* are shown in Figure 2.

### 3.2 Chemical descriptions

Total phenolic content ranged from 1.69 to 14.05 mg GAE g^−1^ FW. Total flavonoid content varied from 0.25 to 2.01 mg QE g^−1^ FW (Table 1). Zhang et al. (2010) reported that the value of total phenolic content in *Z. jujuba* was 32.80 mg GAE g^−1^ DW. Phenolic compounds play an essential role in plants as primary antioxidants or free radical scavengers, and antioxidant activity is due to their redox activity, which plays a key role in the uptake and sterilization of free radicals, quenching singlet and triple oxygen, and decomposition of peroxides (Himesh et al., 2011). Phenolic compounds are a group of antioxidant agents that act as terminators of free radicals, and bioactivity may be due to their ability to chelate metals, inhibit lipoxygenases, and free radical scavenging (Lin et al., 2005; Mallavadhani et al., 2006). Phenolic compounds have also been reported to provide antimutagenic and anticarcinogenic properties in humans when approximately 1.00 g of them is consumed daily through a diet rich in vegetables and fruits (Tanaka et al., 1998).

Total anthocyanin content varied from 5.98 to 76.32 µg CyE g^−1^ FW (Table 1). Anthocyanins are mostly considered as a bioactive compound due to their antioxidant properties. The food industries have explored the potential of anthocyanins to act as natural color, as concerns about the potential side effects of synthetic colors have been increasing (Tarone et al., 2020). Radical scavenging activity ranged from 1.32 to 5.82 mg AsAE g^−1^ FW, while ferric reducing antioxidant power ranged from 35.37 to 93.35 µM FeSO_4_ (Table 1). Brito et al. (2015) reported low antioxidant activity with DPPH and FRAP in *Ziziphus joazeiro*. The most important fruit quality‐related traits and chemical properties of the selected accessions of *Z. jujuba* are shown in Table 3.

**TABLE 3:** The most important fruit quality‐related traits and chemical properties of superior accessions of *Z*. *jujuba* in this investigation

Accession	Yield	Fruit length (mm)	Fruit width (mm)	Fresh fruit weight (g)	Dry fruit weight (g)	Fruit flesh thickness (mm)	Fruit skin color	Fruit flesh texture	DPPH (mg)	FRAP (µmol)	Total anthocyanin (µg)
Tootkan‐50	High	25.48	20.43	6.36	2.59	6.61	Brown	Soft	2.42	54.64	74.26
Tootkan‐97	High	25.90	19.79	5.33	2.24	5.29	Brown	Soft	2.68	52.46	65.61
Tootkan‐99	High	26.28	20.36	5.93	2.23	5.21	Brown	Soft	2.77	58.57	51.99
Tootkan‐61	High	22.46	17.71	5.39	2.17	5.32	Brown	Soft	4.78	77.68	69.41
Tootkan‐55	High	25.69	20.04	5.72	2.12	6.39	Brown	Soft	3.22	51.72	53.83
Tootkan‐59	High	23.82	19.40	4.68	2.07	5.84	Brown	Soft	4.36	75.82	53.04
Tootkan‐67	High	27.20	19.52	5.35	2.07	6.69	Brown	Soft	3.96	67.43	52.65
Tootkan‐22	High	23.02	19.08	4.76	2.07	5.68	Brown	Soft	3.54	66.26	42.96
Tootkan‐96	High	24.43	19.66	4.48	2.06	5.07	Brown	Soft	2.66	52.62	52.65
Tootkan‐66	High	24.66	18.65	5.07	2.04	5.74	Brown	Soft	3.72	58.41	70.20
Tootkan‐75	High	27.35	20.72	5.22	2.04	6.28	Brown	Soft	3.32	61.96	55.62
Tootkan‐49	High	25.43	19.48	5.86	2.02	6.44	Brown	Soft	4.47	62.55	74.65
Tootkan‐38	High	24.01	20.10	5.27	2.01	5.27	Brown	Soft	2.68	42.64	69.28

### 3.3 Correlations between the characters measured

There was a significant and positive correlation between tree height and tree growth vigor (*r* = .54) (not shown). A significant correlation was observed between leaf length and leaf width (*r* = .71) and agreed with the previous results in jujube (Grygorieva et al., 2014; Ivanišová et al., 2017; Khadivi et al., 2021; Mirheidari et al., 2022; Zandiehvakili & Khadivi, 2021). Fruit yield showed positive and significant correlations with tree growth vigor (*r* = .24). Fruit flesh thickness showed significant and positive correlations with fruit length (*r* = .47), fruit width (*r* = .74), fresh fruit weight (*r* = .47), dry fruit weight (*r* = .32), fruit stalk length (*r* = .38), and fruit stalk diameter (*r* = .47), and corresponded with the previous results in jujube (Grygorieva et al., 2014; Ivanišová et al., 2017; Khadivi et al., 2021; Mirheidari et al., 2022; Zandiehvakili & Khadivi, 2021).

Total phenolic content was significantly and positively correlated with fruit taste (*r* = .38), total flavonoid content (*r* = .65), radical scavenging activity (*r* = .48), and ferric reducing antioxidant (*r* = .56) and agreed with the previous findings in jujube (Gao et al., 2012; Zandiehvakili & Khadivi, 2021; Zhang et al., 2010). Total anthocyanin content was significantly and positively correlated with ripening date (*r* = .28), yield (*r* = .22), fresh fruit weight (*r* = .25), and dry fruit weight (*r* = .20). Radical scavenging activity was positively and significantly correlated with fruit taste (*r* = .26) and ferric reducing antioxidant power (*r* = .87) and corresponded with the previous findings in jujube (Gao et al., 2012; Wang et al., 2011; Zhang et al., 2010).

### 3.4 MRA

The MRA showed that total phenolic content was found to be associated with dry fruit weight (standardized beta coefficient, *β* = −.583, *p* < .000), fruit flesh texture (*β* = −.278, *p* < .001), fruit taste (*β* = .279, *p* < .003), and fresh fruit weight (*β* = .363, *p* < .014) (Table 4). Total flavonoid content was associated with fruit flesh color (*β* = −.239, *p* < .017) and fresh fruit weight (*β* = .363, *p* < .014). Also, total anthocyanin content was found to be associated with fresh fruit weight (*β* = .351, *p* < .000). Radical scavenging activity showed significant associations with fruit taste (*β* = .432, *p* < .000), dry fruit weight (*β* = −.321, *p* < .001), and fruit flesh thickness (*β* = .245, *p* < .002). Ferric reducing antioxidant power showed significant associations with fruit taste (*β* = .39, *p* < .000), dry fruit weight (*β* = −.285, *p* < .002), and fruit flesh thickness (*β* = .202, *p* < .004).

**TABLE 4:** The fruit traits associated with chemical properties in *Z. jujuba* as revealed using MRA and coefficients

Dependent character	Independent character	*r*	*r* ^2^	*β*	*t* value	*p* value
Total phenolic content	Dry fruit weight	.377 a	.142	−.583	−4.213	.000
	Fruit flesh texture	.456 b	.208	−.278	−3.271	.001
	Fruit taste	.502 c	.252	.279	3.097	.003
	Fresh fruit weight	.545 d	.297	.363	2.514	.014
Total flavonoid content	Fruit flesh color	.239 a	.057	−.239	−2.437	.017
	Fresh fruit weight	.305 b	.197	.363	2.514	.014
Total anthocyanin content	Fresh fruit weight	.351 a	.123	.351	3.804	.000
Radical scavenging activity	Fruit taste	.503 a	.173	.432	4.324	.000
	Dry fruit weight	.555 b	.232	−.321	−3.877	.001
	Fruit flesh thickness	.591 c	.263	.245	2.234	.002
Ferric reducing antioxidant	Fruit taste	.402 a	.162	.391	3.236	.000
	Dry fruit weight	.455 b	.207	−.285	−2.983	.002
	Fruit flesh thickness	.491 c	.241	.202	2.102	.004

### 3.5 PCA and HCA

The PCA showed that the first 11 components accounted for 77.38% of total variance (Table 5). Branching and branch density were found to be correlated with PC1, accounting for 9.15% of total variance. The PC2 included fruit stalk length, fruit stalk diameter, and fruit stone width, accounting for 8.83% of total variance. Four characters, including fruit width, fresh fruit weight, dry fruit weight, and fruit flesh thickness, formed the PC3, accounting for 8.74% of total variance. In the previous studies, PCA has been used to investigate phenotypic diversity within *Ziziphus* species (Baghazadeh‐Daryaii et al., 2017; Khadivi et al., 2021; Mirheidari et al., 2022; Norouzi et al., 2017; Zandiehvakili & Khadivi, 2021).

**TABLE 5:** Eigenvalues of principal component axes from principal component analysis (PCA) of morphological and chemical characters in the studied accessions of *Z. jujuba*

Character	Component										
	1	2	3	4	5	6	7	8	9	10	11
Habit of tree growth	−0.24	0.14	−0.10	0.19	0.08	0.12	−0.47	0.17	−0.18	0.36	0.20
Vigor of tree growth	0.32	0.00	−0.09	0.05	−0.44	0.03	0.11	0.18	0.58	−0.26	0.25
Height of tree	0.48	−0.03	−0.04	0.11	−0.41	0.07	0.11	−0.13	0.07	−0.25	0.55
Trunk type	−0.24	−0.01	−0.05	−0.10	0.11	0.02	−0.03	0.02	0.03	−0.71^a^	0.05
Density of canopy	0.59	0.00	−0.02	−0.40	−0.38	0.21	0.17	0.05	0.29	−0.04	−0.02
Branching	0.96^a^	0.01	−0.01	−0.05	−0.08	0.09	0.00	−0.01	−0.01	0.17	−0.02
Branch density	0.96^a^	0.01	−0.01	−0.05	−0.08	0.09	0.00	−0.01	−0.01	0.17	−0.02
Branch flexibility	0.13	−0.10	0.23	0.59	0.06	−0.30	−0.06	−0.17	0.16	−0.08	−0.14
Annual shoot length	−0.04	0.06	0.03	0.02	0.83^a^	0.01	0.05	−0.19	0.01	−0.02	−0.06
Thorn on annual shoot	−0.22	0.04	0.05	0.40	0.73^a^	−0.17	−0.14	0.03	0.00	−0.06	−0.03
Length of leaf	0.04	0.03	0.02	−0.13	0.00	0.84^a^	0.11	0.06	0.02	−0.05	0.01
Width of leaf	0.10	−0.04	0.12	−0.04	−0.14	0.84^a^	−0.21	0.02	0.05	−0.01	0.07
Length of petiole	0.10	−0.06	0.09	0.11	0.00	0.83^a^	0.10	−0.07	0.03	0.01	0.00
Thickness of petiole	−0.20	0.14	−0.06	0.03	0.37	−0.07	0.08	−0.46	0.09	0.47	0.27
Ripening date	0.35	−0.27	0.12	−0.35	−0.24	0.15	0.37	0.42	0.12	−0.16	−0.09
Yield	0.03	0.02	−0.01	0.12	−0.08	−0.10	−0.09	0.78^a^	0.01	−0.12	0.03
Length of fruit	−0.04	0.31	0.57	0.01	−0.03	0.20	0.64^a^	−0.08	−0.02	−0.01	−0.01
Width of fruit	−0.17	0.51	0.70^a^	−0.06	0.06	0.06	0.19	−0.13	−0.05	0.07	0.05
Weight of fruit flesh	0.00	0.04	0.92^a^	−0.08	0.03	0.05	0.09	0.13	0.06	0.07	0.07
Dry fruit weight	0.12	−0.02	0.76^a^	−0.24	0.03	0.07	−0.01	0.31	0.09	−0.02	0.11
Length of fruit stalk	0.13	0.70^a^	0.18	−0.11	−0.05	0.15	0.12	0.21	0.04	−0.11	−0.06
Diameter of fruit stalk	−0.13	0.82^a^s	0.12	0.14	0.11	−0.14	0.11	−0.22	−0.08	0.10	0.06
Fruit flesh thickness	−0.14	0.55	0.60^a^	0.14	−0.12	0.21	−0.15	−0.18	−0.11	−0.20	−0.09
Fruit skin color	−0.05	0.00	0.06	−0.09	0.04	0.05	−0.04	0.02	0.91^a^	−0.01	−0.08
Fruit flesh color	0.32	−0.01	0.08	−0.15	−0.44	−0.04	−0.13	−0.06	−0.05	0.53	−0.42
Fruit taste	−0.10	0.03	−0.06	0.18	0.68^a^	−0.04	0.01	0.06	−0.59	−0.18	0.13
Fruit flesh texture	0.16	0.17	0.25	−0.04	0.03	0.20	−0.53	−0.19	0.22	−0.06	−0.31
Fruit stone length	0.09	0.29	0.12	−0.05	0.04	0.06	0.82^a^	0.00	0.02	0.09	0.02
Fruit stone width	0.04	0.91^a^	0.02	−0.07	0.05	−0.09	0.01	−0.02	0.04	0.11	0.01
Weight of fruit stone	0.16	0.42	−0.17	−0.19	0.22	−0.10	0.22	0.24	0.06	0.43	0.18
Total phenolic content	−0.09	−0.07	−0.26	0.59	0.39	−0.39	0.23	0.00	0.00	0.08	0.01
Total flavonoid content	−0.04	0.02	0.17	−0.06	0.01	0.06	−0.01	−0.06	−0.07	0.04	0.79^a^
Total anthocyanin content	−0.12	−0.03	0.19	−0.04	−0.02	0.09	0.09	0.63^a^	0.04	0.16	−0.07
Radical scavenging activity	−0.05	−0.05	−0.16	0.89^a^	0.06	0.16	−0.07	0.12	−0.07	−0.04	−0.04
Ferric reducing antioxidant	−0.15	0.06	−0.15	0.88^a^	0.10	0.03	−0.04	0.04	−0.17	0.12	0.11
Total	3.20	3.09	3.06	3.03	2.89	2.74	2.11	1.93	1.80	1.72	1.52
% of Variance	9.15	8.83	8.74	8.65	8.26	7.82	6.04	5.50	5.16	4.91	4.34
Cumulative %	9.15	17.98	26.72	35.36	43.62	51.44	57.48	62.98	68.13	73.04	77.38

^a^ Eigenvalues ≥0.60 are significant.

**FIGURE 3:**
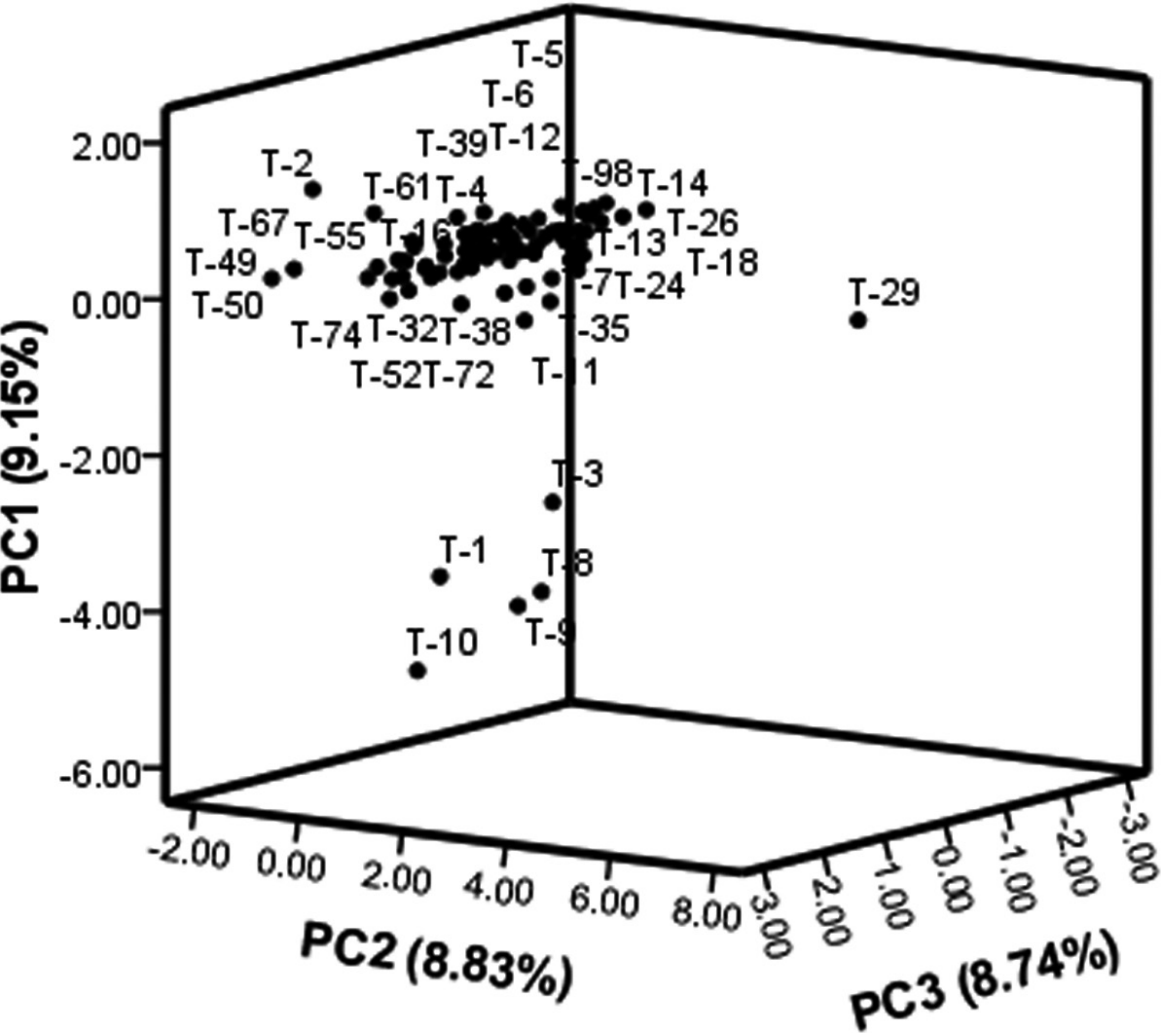
Scatter plot for the studied accessions of *Z. jujuba* based on PC1/PC2/PC3 of combined data of morphological and chemical data. The “T” symbol represents the accessions of Tootkan area

The scatter plot created using PC1/PC2/PC3 showed variations among the accessions (Figure 3). Tootkan‐29 accession showed high differences with other, characterized by low fresh fruit weight, dry fruit weight, and fruit flesh thickness. Also, five accessions, including Tootkan‐1, Tootkan‐3, Tootkan‐8, Tootkan‐9, and Tootkan‐10, formed another group, characterized by moderate fresh fruit weight, dry fruit weight, and fruit flesh thickness. The remaining accessions were placed into the same group.

**FIGURE 4:**
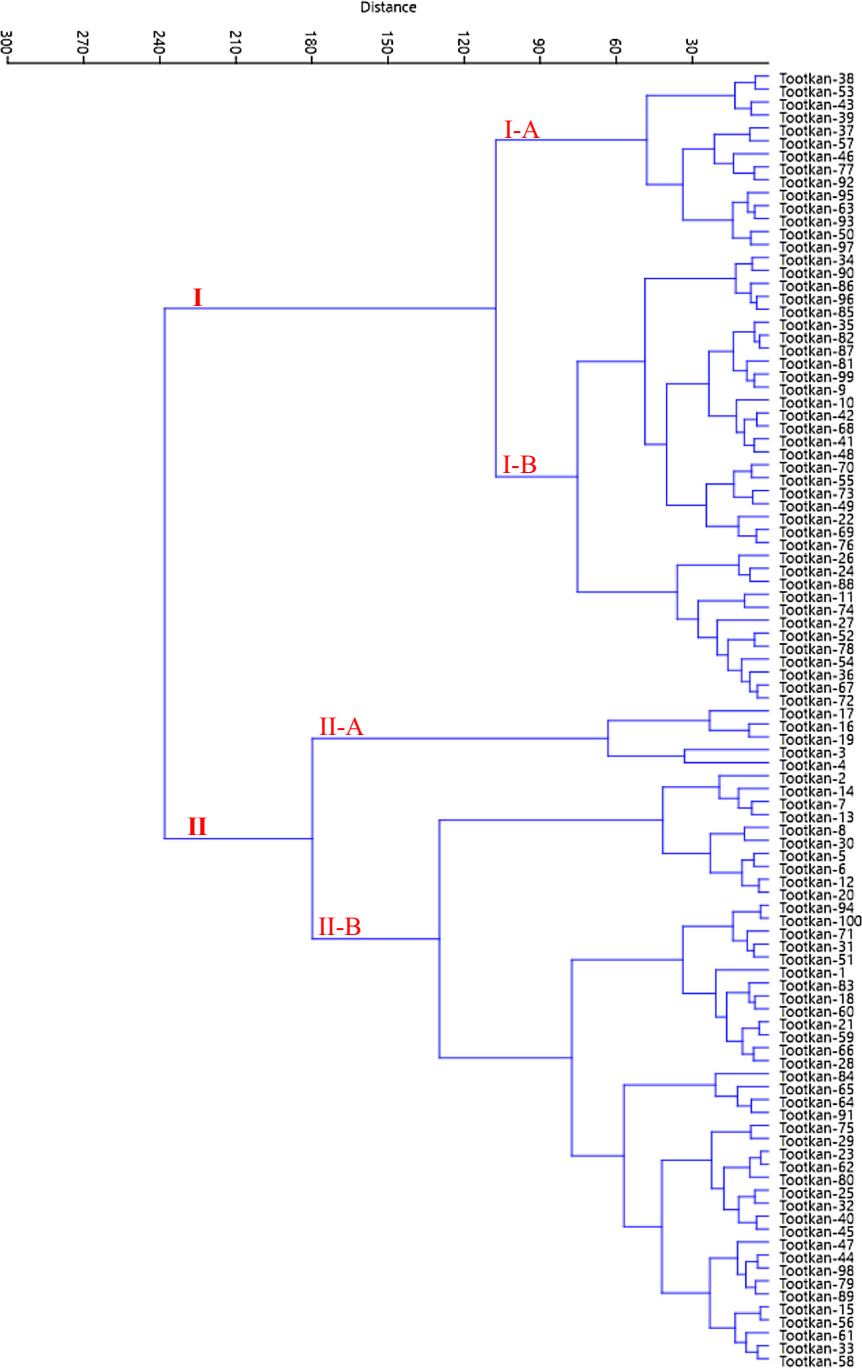
Ward cluster analysis of the studied accessions of *Z. jujuba* based on the combined data of morphological and chemical traits using Euclidean distances

Besides, HCA with Ward dendrogram showed that the accessions were clustered into two major clusters (Figure 4). The first cluster (I) contained 49 accessions, forming two subclusters. Subcluster I‐A included 14 accessions, characterized by the highest values for leaf length, leaf width, petiole length, petiole thickness, and fruit stone weight. Subcluster I‐B included 35 accessions, characterized by moderate values for leaf length, leaf width, petiole length, petiole thickness, and fruit stone weight. The remaining 51 accessions were placed into the second cluster (II), forming two subclusters, characterized by low values for above characters.

## 4 CONCLUSION

Genetic diversity of indigenous genotypes and their related wild accessions is the primary basis for many agricultural research programs, especially breeding programs. Therefore, it is necessary to know the characteristics and potential of these valuable resources collected to use in research programs, so that specialized experts can use them to improve the characters in their programs. The present study showed high diversity in morphological and chemical properties of some jujube accessions. Based on the traits related to fruit quality such as high fruit weight, soft fruit flesh texture, brown fruit skin color, and sweet fruit flavor, as well as in terms of chemical characteristics related to medicinal properties such as higher total anthocyanin content and higher antioxidant activity, 13 accessions, including Tootkan‐50, Tootkan‐97, Tootkan‐99, Tootkan‐61, Tootkan‐55, Tootkan‐59, Tootkan‐67, Tootkan‐22, Tootkan‐96, Tootkan‐66, Tootkan‐75, Tootkan‐49, and Tootkan‐38, were superior. The commercial orchard of those best accessions should be extensively constructed to take advantage of the high yield of *Z. jujuba* as a crop and its medicinal properties.

## CONFLICT OF INTEREST

The authors declare no conflict of interest.

## RESEARCH INVOLVING HUMAN PARTICIPANTS AND/OR ANIMALS

None.

## INFORMED CONSENT

None.

## Data Availability

The data that support the findings of this study are available from the corresponding author upon reasonable request.
